# Identification of cellular senescence-related genes as biomarkers for lupus nephritis based on bioinformatics

**DOI:** 10.3389/fgene.2025.1551450

**Published:** 2025-04-11

**Authors:** Wei Chen, Xiaofang Wang, Guoshun Huang, Qin Sheng, Enchao Zhou

**Affiliations:** ^1^ No.1 Clinical Medical College, Nanjing University of Chinese Medicine (Jiangsu Province Hospital of Chinese Medicine), Nanjing, Jiangsu, China; ^2^ Jiangsu University Key Laboratory of Tonifying Kidney and Anti-senescence, Nanjing, Jiangsu, China; ^3^ Department of Nephrology, Suzhou Hospital of Integrated Traditional Chinese and Western Medicine, Suzhou, Jiangsu, China; ^4^ Department of Nephrology, Suzhou Affiliated Hospital of Nanjing University of Chinese Medicine (Suzhou Hospital of Traditional Chinese Medicine), Suzhou, Jiangsu, China

**Keywords:** lupus nephritis, cellular senescence, biomarker, weighted gene Co-expression network (WGCNA), machine learning

## Abstract

**Background:**

Lupus nephritis (LN) is one of the most common and severe complications of systemic lupus erythematosus with unclear pathogenesis. The most accurate diagnosis criterion of LN is still renal biopsy and nowadays treatment strategies of LN are far from satisfactory. Cellular senescence is defined as the permanent cell cycle arrest marked by senescence-associated secretory phenotype (SASP), which has been proved to accelerate the mobility and mortality of patients with LN. The study is aimed to identify cellular senescence-related genes for LN.

**Methods:**

Genes related to cellular senescence and LN were obtained from the MSigDB genetic database and GEO database respectively. Through differential gene analysis, Weighted Gene Go-expression Network Analysis (WGCNA) and machine learning algorithms, hub cellular senescence-related differentially expressed genes (CS-DEGs) were identified. By external validation, hub CS-DEGs were further filtered and the remaining genes were identified as biomarkers. We explored their potential physiopathologic function through GSEA.

**Results:**

We obtained 432 genes related to cellular senescence, 1,208 differentially expressed genes (DEGs) and 840 genes in the key gene module related to LN, which were intersected with each other for CS-DEGs. Subsequent Machine learning algorithms screened out six hub CS-DEGs and finally three hub CS-DEGs, ALOX5, PTGER2 and PRKCB passed through external validation, which were identified as biomarkers. The three biomarkers were enriched in “B Cell receptor signaling pathway” and “NF−kappa B signaling pathway” based on GESA results.

**Conclusion:**

This study explored the potential relationship between cellular senescence and LN, and identified three biomarkers ALOX5, PTGER2, and PRKCB playing key roles in LN, which will provide new insights for the diagnosis and treatment of LN.

## 1 Introduction

Lupus nephritis (LN) is one of the most common and severe complications of systemic lupus erythematosus (SLE), marked by heterogeneous clinical presentation ranging from slight urinary abnormalities to prominent nephrotic syndrome or rapidly progressive renal insufficiency (Moroni et al., 2016; Anders et al., 2020). So far, the most accurate diagnosis criterion of LN is still renal biopsy, which helps recognize kidney disease subtype, justify disease activity and guide therapeutic decisions. But renal biopsy is accompanied by histologic risks of hemorrhage, infection and arteriovenous fistula. Apart from the diagnostic limitations, although multiple treatment strategies of LN have been in continuous optimization, the therapeutic goal is not always achieved as expected for the drug toxicity on multi-organ, low response of treatment and relapses of lupus nephritis (Kronbichler et al., 2019). Therefore, new targets for LN diagnosis and therapy are imperatively needed.

Cellular senescence is defined as the permanent cell cycle arrest marked by senescence-associated secretory phenotype (SASP), which means the abnormal production of chemokines, cytokines, growth factors, and proteases (Gao et al., 2019). Cellular senescence along with SASP is documented to serve crucial functions in multiple inflammatory disorders and chronic diseases including diabetes, cardiac diseases, osteoarthritis, neurodegenerative disease and cancer (He and Sharpless, 2017; Mehdizadeh et al., 2022; Zhang et al., 2022). In recent years, the relationship between LN and cellular senescence has induced more concern, providing a novel perspective for the pathology and administration of LN. Evidence showed that DNA damage and excessive production of ROS caused by cellular senescence will accelerate the mobility and mortality of patients with LN (Gao et al., 2019). Several experiments proved renal damage of cellular senescence in lupus mice, and suppressive therapy targeting the accumulation of cellular senescence is beneficial for remission of LN (Tilman et al., 2023).

However, there is rare research exploring the function of cellular senescence in the context of LN from molecular or cellular perspectives. Hence, we focus on the cellular senescence related to LN and utilize bioinformatic ways to find genes associated with cellular senescence and LN from the Gene Expression Omnibus (GEO) database. Then machine learning algorithms were used to screen out hub genes with talent predictive value. In addition, we also employed Gene Set Enrichment Analysis (GSEA), immune filtration analysis and transcription network analysis to understand the potential roles of genes related to cellular senescence in the pathogenic progress of LN, with a desire to inspire new diagnosis or treatment strategies for optimizing clinical management of LN.

## 2 Materials and methods

### 2.1 Data collection

**FIGURE 1:**
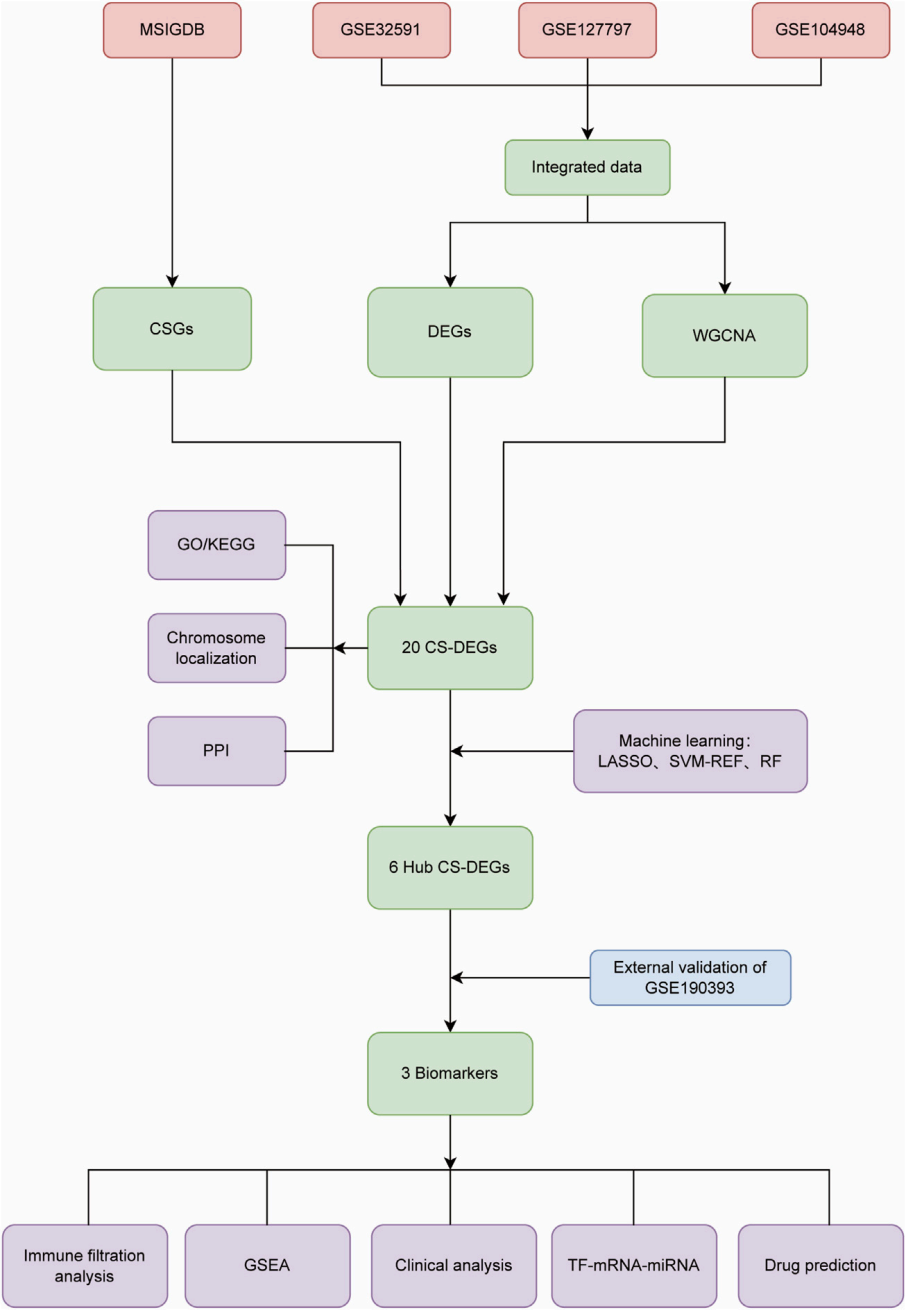
The flow diagram of the study.

We searched the National Center for Biotechnology Information Gene Expression Omnibus (GEO) database (http://www.ncbi.nih.gov/geo/) for Lupus nephritis gene expression profile data. GSE32591 (32LN samples and 14 controls, GPL14663), GSE127797 (41 LN samples, GPL24299) and GSE104948 (32 LN samples and 21 controls, GPL24120 and GPL22945) were selected for training, and GSE180393 (15 LN samples and nine controls, GPL19983) was used as a validation set. All of the kidney tissue samples above were acquired from glomerulus, rather than other tissue specimens. We employed the “sva” R package to integrate gene expression information of the three datasets based on different platforms in order to remove batch effects. After data pre-progressing, the training set contained 105 LN patients and 35 healthy controls. Cellular Senescence-related genes (CSGs) were obtained from the MSigDB (Molecular Signatures Database) genetic database (https://www.gsea-msigdb.org/gsea/msigdb), as shown in Figure 1.

### 2.2 Identification of differential expression genes in LN

The “limma” R package was utilized to identify differentially expressed genes (DEGs) between LN patients and healthy controls in the training set, with the threshold set as |log2FC| > 0.5 and p < 0.05. Totally 1,208 DEGs were screened and the result was presented in the form of heatmap and volcano ploy by the “heatmap” package and the “ggplot2” package, respectively.

### 2.3 WGCNA

We performed Weighted Gene Go-expression Network Analysis (WGCNA) in advantage of the “WGCNA” package. In the beginning, variance of genes expression levels in the training set was calculated and genes with the top 25% variance were extracted for subsequent analysis. To eliminate outliers of the samples, we constructed a sample tree via the “hclust” function. The inclusion criterion was set as tree height below 52 and three samples were removed. We created an adjacency matrix to define the connection power between each two genes, with the best soft thresholding decided to be seven through the “pickSoftThreshold” method, where the corresponding R2 was 0.9. Then the topological overlap measure (TOM) was used to assess the proximity between genes for the construction of a scale-free topology network, which combined the adjacency of two genes and the connection strengths these two genes shared with another gene. We categorized genes into different gene modules according to their interconnection by a dynamic tree-cutting algorithm, which means that genes in the same module exhibited similar expression patterns. Each module contained at least 30 genes and genes of cluster tree height below 0.25 were integrated into one module for the consideration that their expression characteristics were parallel. Finally, we calculated gene significance (GS) and module membership (MM) to evaluated the association strength between gene modules and the clinical feature, which defined as LN in our research. The module with the strongest relevance to LN was identified to be a key module.

### 2.4 Identification of CS-DEGs and functional enrichment analysis

The intersection of CSGs, DEGs and genes in the key module were judged as CS-DEGs linked to LN and the analyzing progress was visual in the manner of a Venn plot. After the acquisition of CS-DEGs, we used the “RCircos” package to localize each gene of the CS-DEGs set in human chromosome. Based on the STRING database (https://www.string-db.org/), protein-protein interaction (PPI) network was analyzed to exhibit the interaction within the CS-DEGs. Moreover, Gene Ontology (GO) and Kyoto Encyclopedia of Genes and Genomes (KEGG) enrichment analyses were performed through the “clusterProfiler” package to speculate possible function of CS-DEGs in LN.

### 2.5 Identification of hub CS-DEGs by machine learning

We used three machine learning algorithms including Least absolute shrinkage and selection operator (LASSO) regression, Support Vector Machine-Recursive Feature Elimination (SVM-RFE) and Random Forest (RF) analysis to identify hub CS-DEGs. LASSO regression is a form of regularization designed to limit prediction error and enhance the interpretability of the statistical model (Ranstam and Cook, 2018). SVM-RFE selects vital genes by delaminating variables with the lowest weights based on their importance ranking (Cortes and Vapnik, 1995; Sanz et al., 2018). RF is a meta estimator designed to fit a number of decision tree classifiers on various sub-samples of the dataset aiming to reduce the number of variables while maintaining computational efficiency (Breiman, 2001). We used the “glmnet” package, the “e1071” package and the “randomForest” package to discern hub CS-DEGs that overlapped across these three machine learning algorithms.

Specifically, for LASSO regression, we employed the “glmnet” package and trained the model using 10-fold cross-validation to determine the optimal regularization parameter (λ). The best λ was selected based on the minimum mean cross-validated error. For SVM-RFE, the “msvmRFE” package was utilized to perform 10-fold cross-validation, ensuring robustness in feature selection. To enhance reproducibility, the process was repeated multiple times with a fixed random seed. The Random Forest model was constructed using 1,000 trees, with the optimal “mtry” parameter determined by evaluating the out-of-bag (OOB) error across different values. Besides, feature importance was assessed using the Mean Decrease in Accuracy and the Mean Decrease in Node Impurity. These systematic steps ensured that the selected features were consistently robust across the different machine learning methods.

Additionally, in order to access the importance of hub CS-DEGs, receiver operating characteristic (ROC) curves were drew by the “pROC” package to show the ability of each gene to predict LN in the training set, along with the validation set (GSE180393). The magnitude of area under the ROC curve (AUC) and the consistency of genes expression pattern in the training and validation set were the criteria for choosing hub CS-DEGs as potential biomarkers.

### 2.6 GSEA analysis

Gene Set Enrichment Analysis (GSEA) is an analytical technique for interpreting gene biological function and clinical connection with LN based on gene expression matrix. To explore underlying biological function of biomarkers, we conducted single-gene GSEA enrichment analysis to uncover latent KEGG pathways through the “clusterProfiler” package. The p value <0.05 was used as the cut-off threshold.

### 2.7 Immune cell infiltration analysis

We calculated the abundance of 22 immune cell within LN samples and healthy controls, and investigated statistical differences between the two groups in the “CIBERSORT” package. Furthermore, we analyzed the correlation between gene expression and the abundance of 22 immune cell infiltration to explore the influence of biomarkers to immune microenvironment in the context of LN.

### 2.8 Construction of “TF-miRNA-gene” network and prediction of potential drugs

We downloaded interaction information of transcription factors (TFs) and miRNAs associated with biomarkers from the NetworkAnalyst database (https://www.net-workanalyst.ca/), which renders comprehensive gene expression data verified by experimental studies as well as computational predictions. The Cytoscape software was utilized to complete the mapping of the “TF-miRNA-gene” network.

The DGIdb database (https://www.dgidb.org/) is a public resource consolidates disparate data sources describing drug-gene interactions and gene druggability (Cannon et al., 2024). We searched the website to discover drugs or small organic compounds aiming at biomarkers, which were potential to be novel LN therapeutic drugs.

### 2.9 Clinical analysis

For the sake of the relationships between biomarkers and clinical features and pathological types of LN, the Nephroseq database (https://nephroseq.org/resour-ce/main.html), which provides extensive information on kidney disease burden and molecular mechanisms, was used for analysis.

## 3 Results

### 3.1 Data acquisition and processing

**FIGURE 2:**
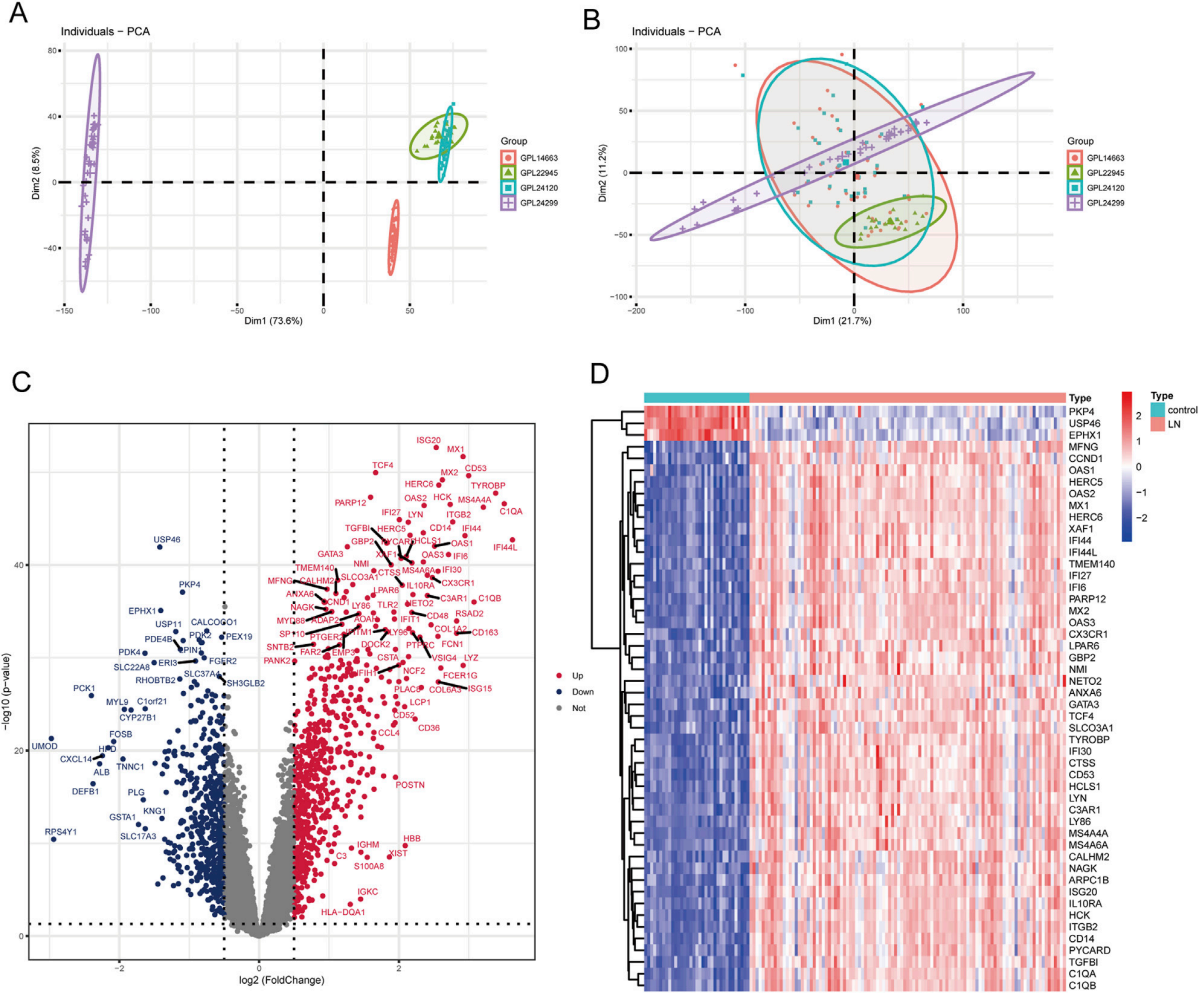
Identification of DEGs in LN. **(A)** PCA scatter plot of the training set before batch correction. **(B)** PCA scatter plot of the training set after batch correction. **(C)** Volcano plot of DEGs between LN and normal groups. **(D)** Heatmap of the top 50 DEGs.

Using the “sva” package, batch effects were eliminated in training set merged by GSE3259, GSE127797 and GSE104948, as shown in Figures 2A, B. According to expression profile data derived from the training set containing 10,490 genes, 1,208 DEGs between LN and healthy group were obtained, among which 660 genes were upregulated and 548 were downregulated, as shown in the volcano map and heatmap (Figures 2C, D). Besides, 432 genes relevant to cellular senescence (CSGs) were found from the MSigDB database.

### 3.2 Identification of LN-related gene modules

**FIGURE 3:**
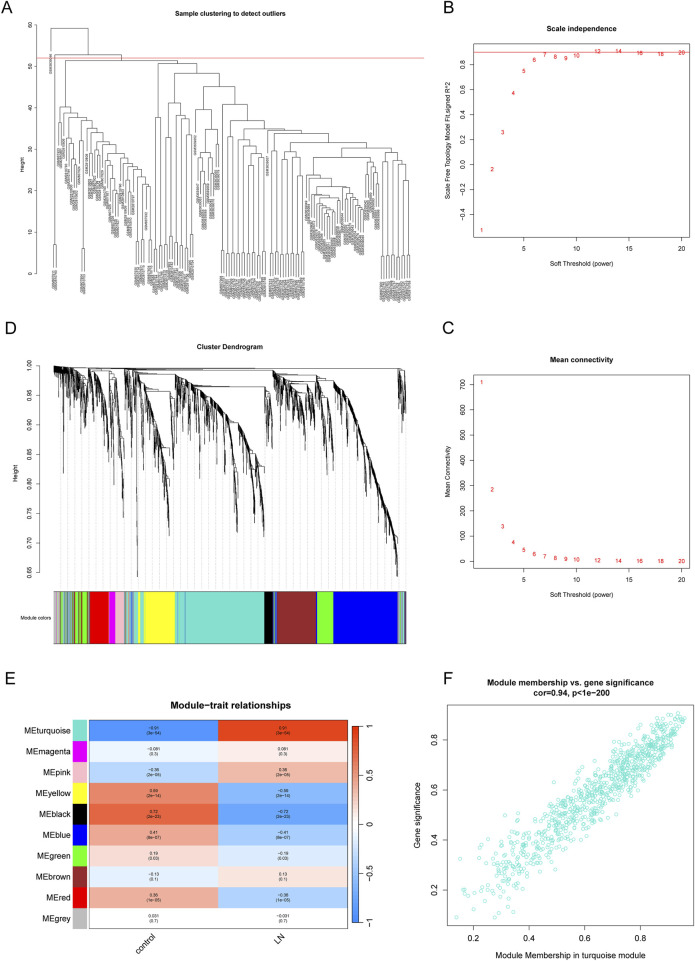
Identification of gene modules related to LN by WGCNA. **(A)** Sample clustering dendrogram of 137 samples in the training set with three outliers eliminated. **(B, C)** The scale-free fit index and the mean connectivity for different soft-thresholding powers (β). **(D)** Dendrogram of genes clustered *via* the dissimilarity measure (1-TOM) and hierarchical clustering. **(E)** Heatmap of the correlation between module genes and the disease status of LN. **(F)** Scatter plot of gene significance (GS) *versus* module membership (MM) of the turquoise module.

WGCNA was performed to dig out key gene modules related to LN. The sample dendrogram (Figure 3A) revealed the sample clustering results, and three outliers were deleted for the robustness of analysis. The soft threshold was chosen as seven to produce higher similarity with a scale-free network as elucidated by the results of the scale-free topology model fit and mean connectivity (Figures 3B, C). The gene hierarchy clustering dendrogram (Figure 3D) showed the distribution of modules. A total of 10 modules were obtained through average hierarchical clustering and dynamic tree clipping, as exhibited in the heatmap (Figure 3E). MEturquoise, MEpink, MEyellow, MEblack, MEblue and MEred possessed statistically significant relationship with LN. Among the 10 modules, the turquoise module consisting of 840 genes was selected as key gene module for the reason that it displayed the highest correlation with LN (a correlation coefficient of 0.91 and a p-value of 3e-5). Meanwhile, a significant correlation was noted between gene significance (GS) and module membership (MM) in the turquoise module, with a correlation coefficient of 0.94 and a p-value of 1e-200 (Figure 3F). Genes in the turquoise module were identified as key genes for further study.

### 3.3 Identification of CS-DEGs and functional enrichment analysis

**FIGURE 4:**
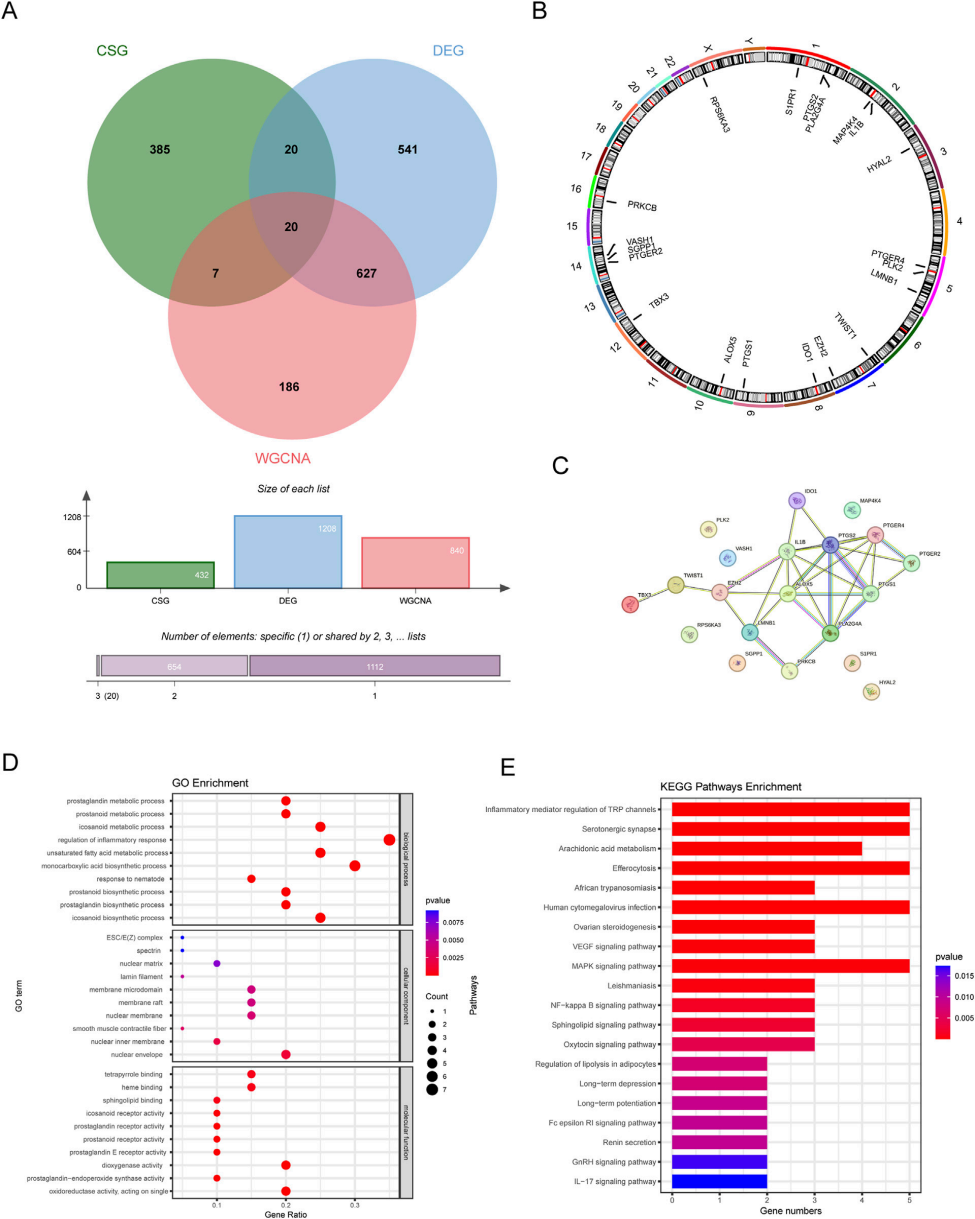
The identification and function analysis of CS-DEGs. **(A)** Venn diagram showing 20 CS-DEGs in LN that overlapped DEGs, key module genes, and CSGs. **(B)** Chromosome localization circles of CS-DEGs. **(C)** PPI network of CS-DEGs. **(D)** GO results **(E)** KEGG analysis results.

We plotted a Venn diagram to show the logical relation between DEGs, CSGs and genes in the key module (Figure 4A). A total of 20 genes overlapping above gene sets were emerged as CS-DEGs, which consisted of PLK2, TBX3, TWIST1, VASH1, HYAL2, EZH2, LMNB1, MAP4K4, RPS6KA3, IDO1, IL1B, ALOX5, PLA2G4A, PTGER2, PTGER4, PTGS1, PTGS2, PRKCB, S1PR1 and SGPP1. Figure 4B shows the location of 20 CS-DEGs on 23 human chromosomes. PPI network analysis (Figure 4C) revealed the gene PTGS2 and IL1B connected to the most interdependent proteins and functioned as central roles, followed by ALOX5, PTGER4, PTGS1 and PLA2G4A.

GO enrichment analysis revealed that CS-DEGs may participate in prostanoid metabolic process, regulation of inflammatory response and monocarboxylic acid biosynthetic process, as shown in Figure 4D. As for KEGG pathway analysis, the top 20 enriched pathways of CS-DEGs involved Inflammatory mediator regulation of TRP channels, Serotonergic synapse, Arachidonic acid metabolism, VEGF signaling pathway, MAPK signaling pathway and NF-kappa B signaling pathway, more details seen in Figure 4E.

### 3.4 Obtainment of hub CS-DEGs by machine learning

**FIGURE 5:**
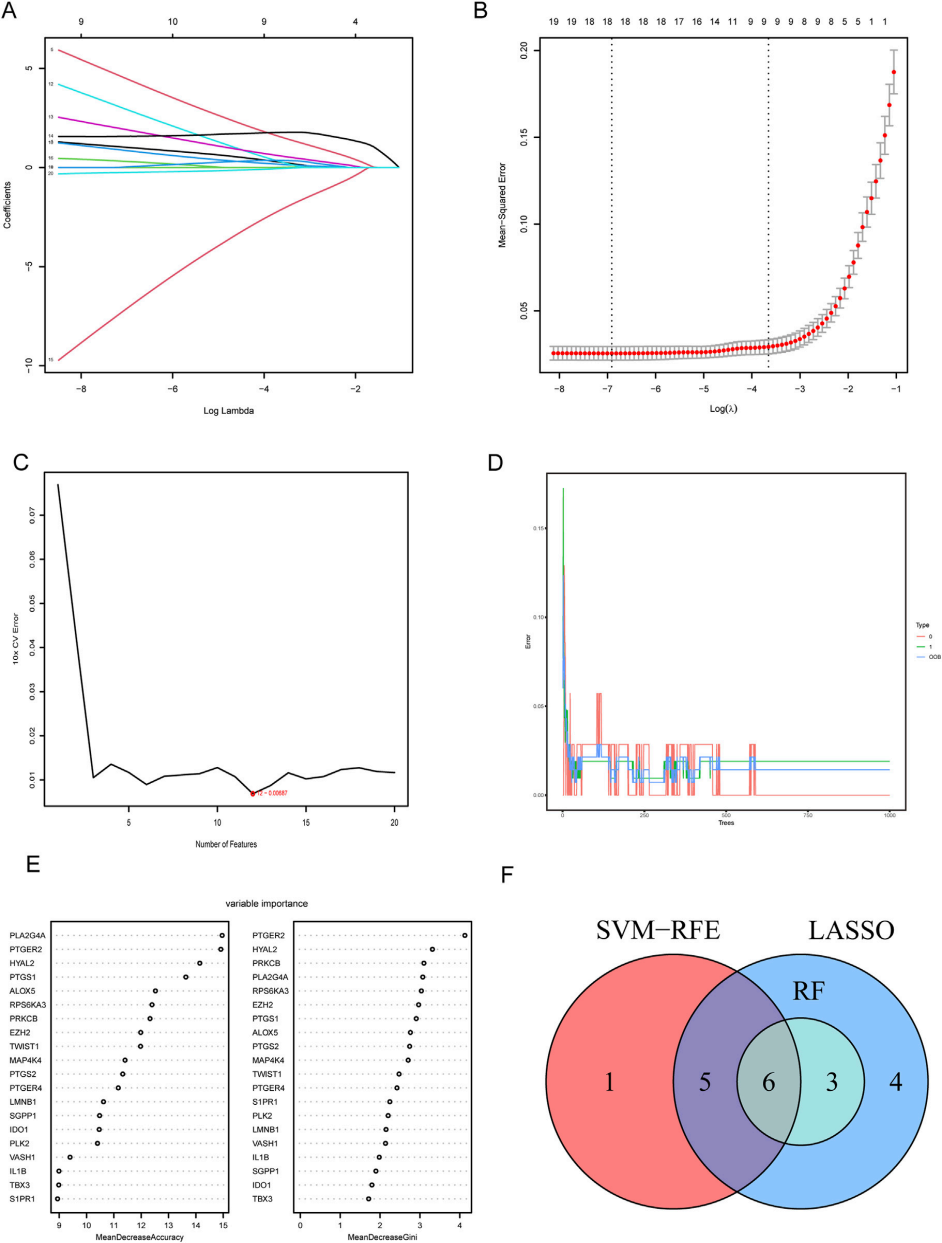
Screening hub CS-DEGs by machine learning. **(A)** LASSO regression of 10 hub genes. **(B)** Cross validation of parameter selection in LASSO regression. **(C)** The important feature selection graph obtained by SVM-RFE algorithm. **(D)** RF algorithm illustrating the relationship between the number of trees and error rate. **(E)** Ranking of genes based on their relative importance through the RF algorithm. **(F)** Venn diagram showing the hub genes shared by SVM-RFE, LASSO and RF algorithms.

To determine the hub CS-DEGs, we employed three machine learning methods. First, we performed the lasso regression analysis, an algorithm invol¬ving an L1 penalty to screen out genes strongly associated with LN. With the optimal λ value, 18 genes (PLK2, TBX3, VASH1, HYAL2, EZH2, LMNB1, MAP4K4, RPS6KA3, IDO1, ALOX5, PLA2G4A, PTGER2, PTGER4, PTGS1, PTGS2, PRKCB, S1PR1 and SGPP1) were obtained (Figures 5A, B). Next, SVM-RFE analysis selected 12 genes (PTGER4, YAL2, PTGER2, ALOX5, PLA2G4A, PTGS1, IDO1, LMNB1, IL1B, SGPP1, PLK2 and PRKCB) as highly correlated with LN (Figure 5C). Last, the RF algorithm generated a rank list about Mean Decrease Accuracy and Mean Decrease Gini of CS-DEGs (Figures 5D, E). Genes belonged to the Top10 Mean Decrease Accuracy ranking list and the Top10 Mean Decrease Gini ranking list were judged as important genes related to LN, which made up 9 (PLA2G4A, PTGER2, HYAL2, PTGS1, ALOX5, RPS6KA3, PRKCB, EZH2 and MAP4K4). We handled the genes obtained from three algorithms with the “VennDiagram” package and the intersection of them were identified as hub CS-DEGs, including HYAL2, PTGER2, ALOX5, PLA2G4A, PTGS1, PRKCB (Figure 5F).

**FIGURE 6:**
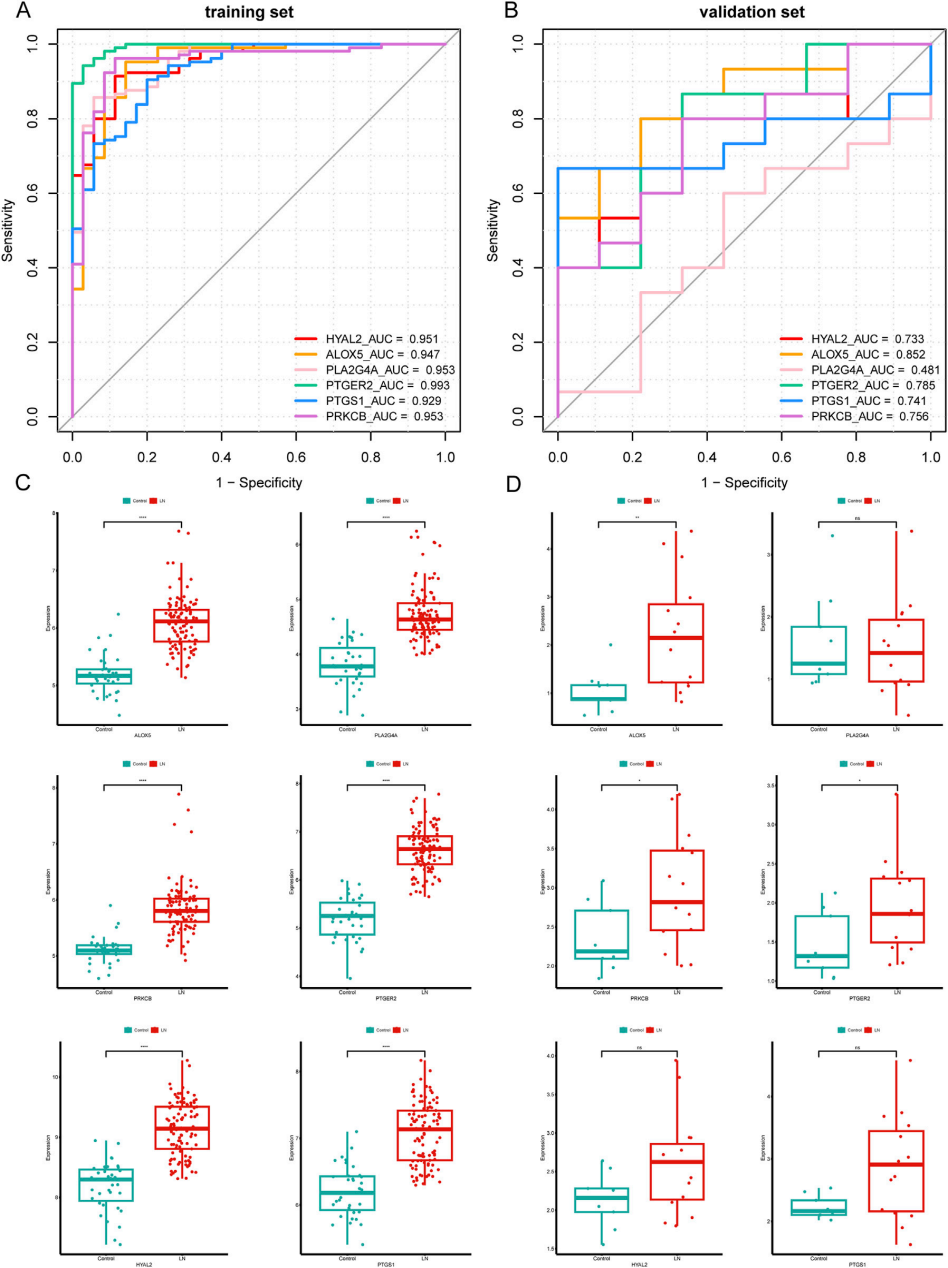
Validation of six hub CS-DEGs. **(A, B)** ROC curves of the six hub genes in the training set and the external validation set. ALOX5, PRKCB, and PTGER2 demonstrated strong diagnostic values for LN in the external validation set (AUC> 0.75). **(C, D)** The expression pattern of the six hub genes in the external validation set.

The predictive power for LN of six hub CS-DEGs demonstrated outstanding for their AUC all above 0.9 in the training set (Figure 6A). Then we drew ROC curves of each gene in the external independent validation set and found three genes of them (ALOX5, PTGER2, PRKCB) retained dominant predictive ability, with the AUC of which above 0.75 (Figure 6B). Besides, box plots (Figures 6C, D) illustrated that the expression pattern of ALOX5, PTGER2, PRKCB in the validation set was entirely consistent with the training set because all of these genes were upregulated in the LN samples. In conclusion, ALOX5, PTGER2, PRKCB were identified as biomarkers related to LN.

### 3.5 GSEA analysis

**FIGURE 7:**
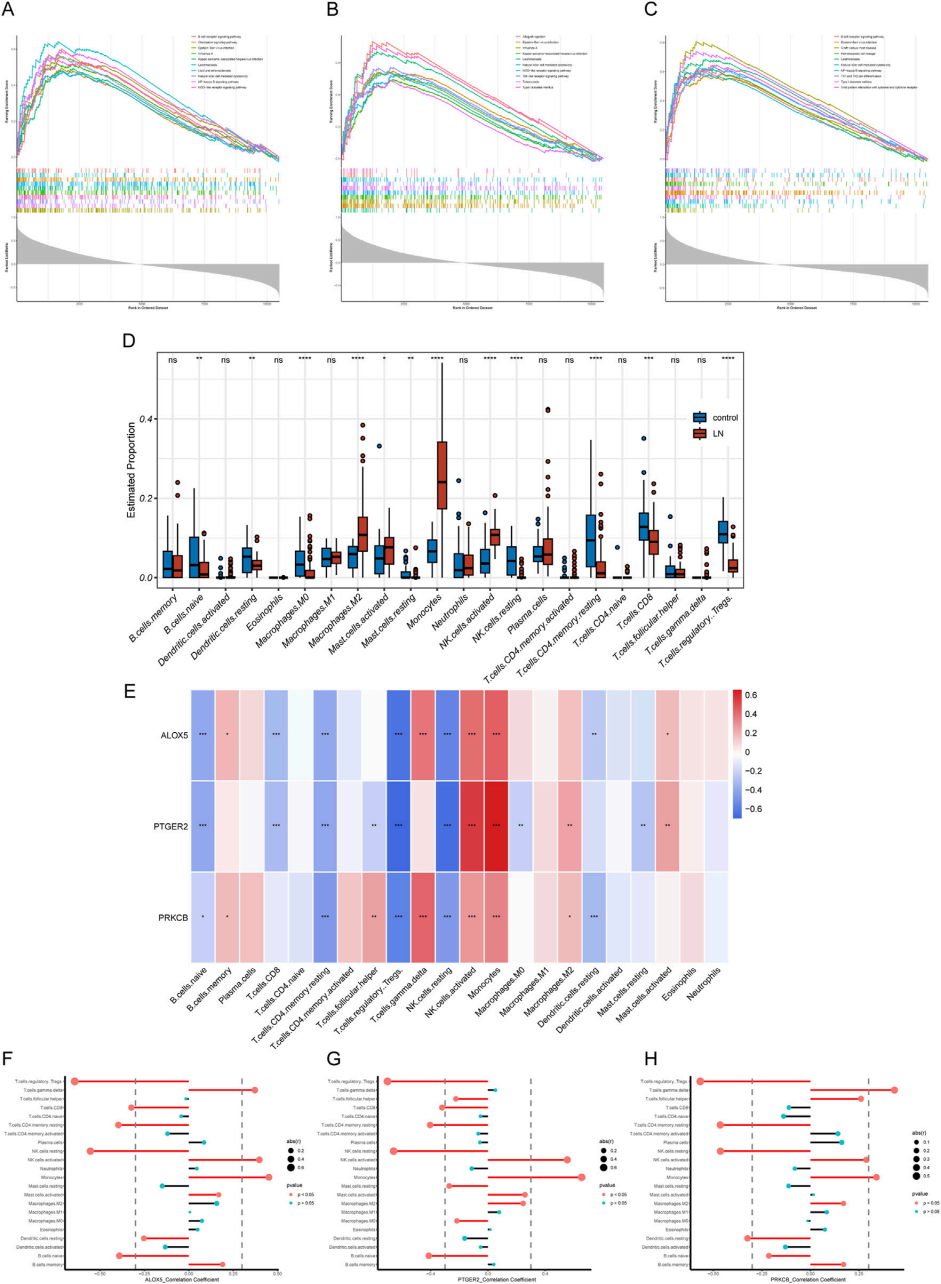
GSEA and immune filtration analysis results. **(A–C)** Single-gene GSEA of biomarkers (ALOX5, PTGER2, and PRKCB). **(D)** Abundances of twenty-one immune cells differed significantly in LN. **(E)** Heatmap of correlation analysis of biomarkers and twenty-one kinds of immune cells **(F–H)** Lollipop plots of correlation analysis of biomarkers and twenty-one kinds of immune cells.

To explore the potential physiopathologic function of ALOX5, PTGER2 and PRKCB in LN, we implemented single gene GSEA. The results were shown in Figures 7A–C, reflecting enrichment functional pathways of each gene by comparing gene expression data in descending order with predefined gene sets. Based on GSEA results, ALOX5 was relevant to B Cell receptor signaling pathway, Chemokine signaling pathway, Lipid and atherosclerosis, Natural killer cell mediated cytotoxicity, NF−kappa B signaling pathway and NOD−like receptor signaling pathway. PTGER2 was relevant to Natural killer cell mediated cytotoxicity, NOD−like receptor signaling pathway and Toll−like receptor signaling pathway. PRKCB was relevant to B Cell receptor signaling pathway, Natural killer cell mediated cytotoxicity, NF−kappa B signaling pathway, Th1 and Th2 cell differentiation and Viral protein interaction with cytokine and cytokine receptor.

### 3.6 Immune cell infiltration analysis

LN is a multi-organ disease with a diverse pathogenesis characterized by immune system dysfunction. In order to find the relationship between expression level of hub CS-DEGs and immune cell abundance, we carried out immune cell infiltration analysis through the “CIBERSORT” package. First, we studied proportions of immune cells in LN group and healthy group. The box plot (Figure 7D) illustrated that 12 out of 22 immune cells expressed differentially in LN group, including naive B Cells, resting dendritic cells, Macrophages M0, Macrophages M2, resting Mast cells, activated Mast cells, Monocytes, resting NK cells, activated NK cells, CD4 memory resting T Cells, CD8 T Cells and regulatory T Cell. The results of single gene CIBRSORT analysis uncovered that ALOX5, PTGER2 and PRKCB presented similar immune cells expressing pattern (Figures 7E–H). They shared positive correlation with activated NK cells (p < 0.001), Monocytes (p < 0.001) and negative correlation with resting NK cells (p < 0.001), regulatory T Cells (p < 0.001), Resting memory CD4 T Cells (p < 0.001) and naive B Cells (p < 0.05). In addition, both ALOX5 and PRKCB were positively related to gamma delta T Cell (p < 0.001).

### 3.7 Construction of regulatory network and prediction of potential drugs

**FIGURE 8:**
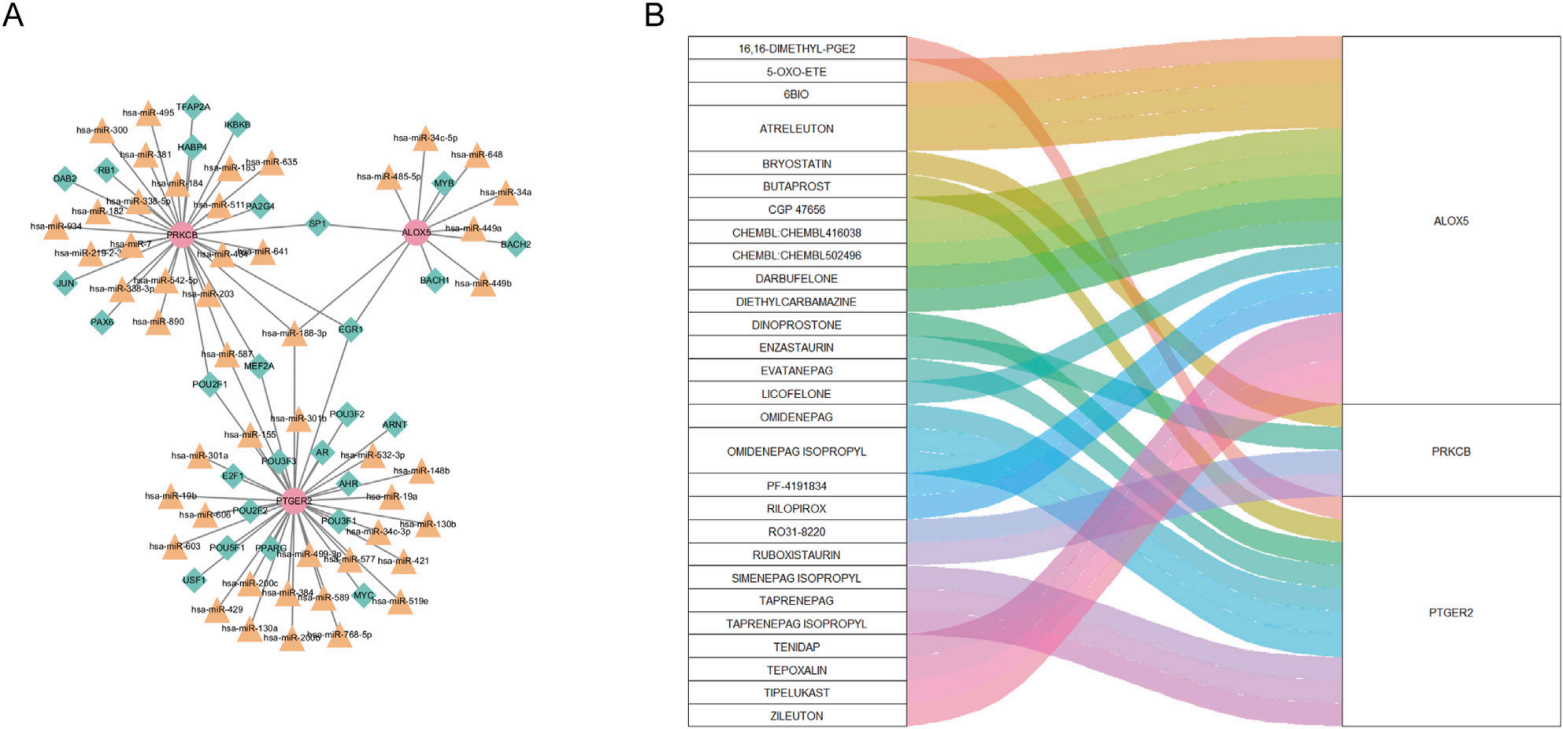
**(A)** “TF-miRNA-gene” network presenting the regulatory mechanisms of biomarkers. **(B)** The relationship between biomarkers and drugs predicted from the public database.

As can be seen from Figure 8A, the “TF-miRNA-gene” network with 78 nodes and 83 edges manifested interaction between TF, miRNA and three biomarkers. In the network, EGR1 and Has-miR-188–3p regulated expression of ALOX5, PTGER2 and PRKCB. Based on the DGIdb database, we gained 66 drugs targeting at any of the hub CS-DEGs. The top 30 drugs ranking on interaction score were presented in Figure 8B.

### 3.8 Clinical analysis

**FIGURE 9:**
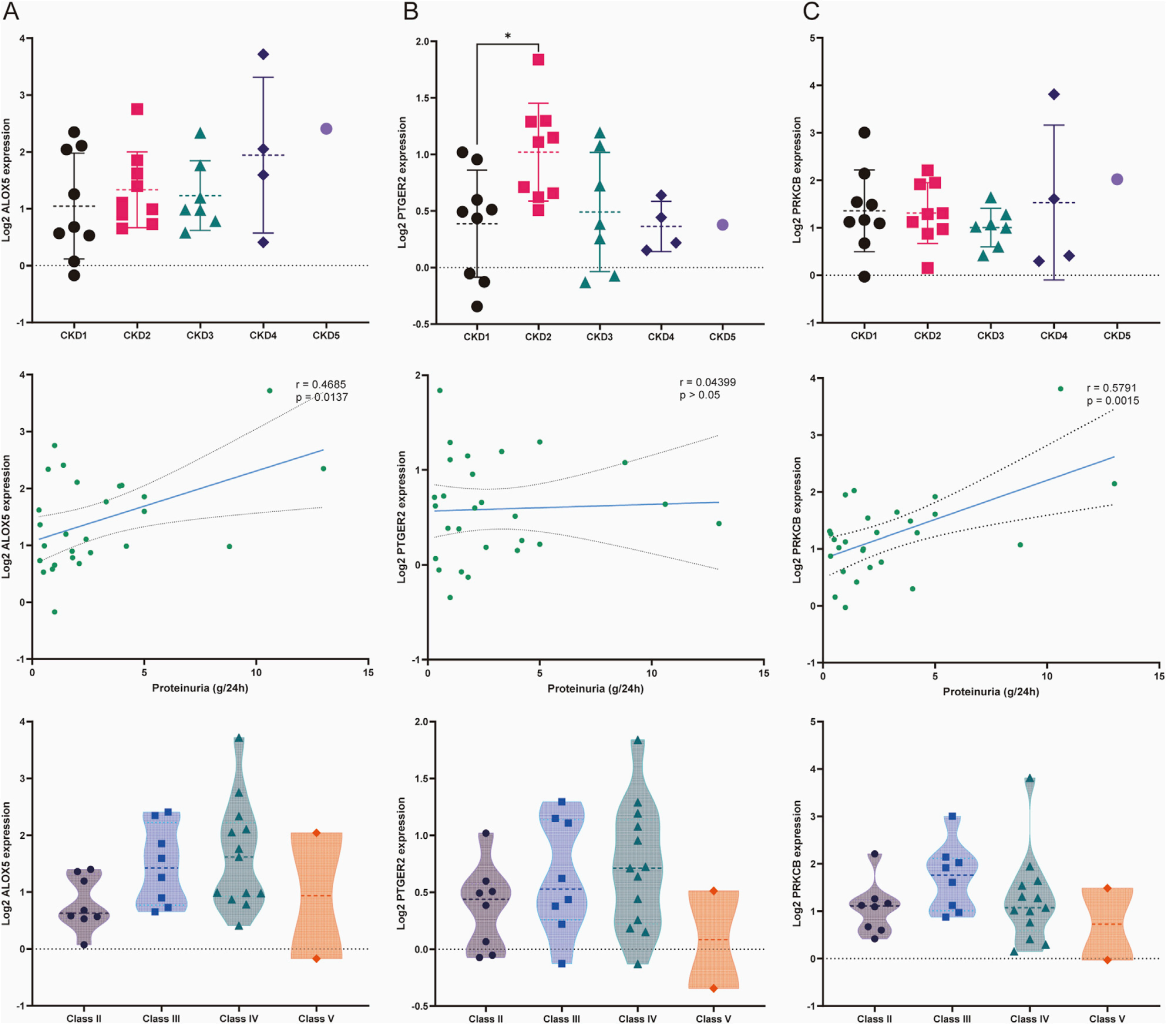
Relationships between the expression of biomarkers and stage of chronic kidney disease (CKD), proteinuria and LN pathological classification. **(A)** Relationship between the expression of ALOX5 and three variables: stage of CKD, proteinuria and pathological classification. **(B)** Relationship between the expression of PTGER2 and three variables: stage of CKD, proteinuria and pathological classification. **(C)** Relationship between the expression of PRKCB and three variables: stage of CKD, proteinuria and pathological classification.

The results of the correlation analysis in the Nephroseq database were exhibited in Figures 9A–C. PTGER2 expression levels were significantly elevated in patients of CKD stage 2 compared with that of CKD stage 1. The relationships between other biomarkers and CKD stages were not statistically significant. When it comes to proteinuria levels, both ALOX5 and PRKCB were positively related to proteinuria levels. But the relationship between PTGER2 and proteinuria did not reach statistical significance. We also attempted to analyze the relationship between biomarkers and LN pathological types. In spite of the lack of statistical significance of the differences between biomarker expression in diverse LN types, there was a tendency that ALOX5 and PRKCB were highly expressed in patients of class Ⅱ.

## 4 Discussion

LN is a common organ manifestation of SLE with ambiguous etiology and almost half of SLE patients suffer from LN within 5 years of an SLE diagnosis (Anders et al., 2020). Currently available treatment of LN contains nonsteroidal anti-inflammatory drugs (NSAIDs), antimalarial drugs, corticosteroids, immunosuppressants and biologics. But clinical efficacy of existing treatment strategies are far from satisfactory, which highlights the request for novel insights for the pathology and management of LN.

Cellular senescence is an irreversible progress characterized by permeant arrest of cell cycle and the development of a multi-component senescence-associated secretory phenotype (SASP), which plays a crucial role in chronic disorders and non-infectious diseases (Spinelli et al., 2023). Different types of glomerular nephritis are demonstrated at the nexus of cellular senescence markers (Valentijn et al., 2018). Several studies reported that in mesenchymal stem cells (MSCs) from SLE patients, signaling pathways including PI3K/Akt, PTEN/Akt, JAK-STAT, p53/p21, and Wnt/beta-catenin are activated in the development of the senescence phenotype (Li et al., 2021). MRL/lpr mice with severe proteinuria displayed increased glomerular expression of senescence-associated β-galactosidase (SA-β-Gal), positively related to urinary protein levels and expression of α-SMA, which resulted in the postulation that accelerated senescence of cells induced glomerular injury in LN (Yang et al., 2018). Dihydroartemisinin (DHA) was proven beneficial in ameliorating the symptoms in pristane-induced lupus mice including renal injury *via* protecting myeloid-derived suppressor cells (MDSCs) from senescence (Li et al., 2019). Similarly, in LN patients, the biomarker of cellular senescence p16INK4a highly expressed and was significantly associated with lower estimated glomerular filtration rate and 5 years post-treatment (Tilman et al., 2023). Based on that, it is reasonable to speculate that cellular senescence makes contributions to the pathogenesis of LN.

This study utilized bioinformatic strategies to analyze the relationship between LN and cellular senescence. We obtained 1,208 DEGs related to LN from the microarray datasets. Then 20 CS-DEGs were identified through overlap calculation of DEGs related to LN, CSGs and WGCNA genes. We conducted GO and KEGG pathway enrichment analysis to interpret possible function behind the pathology of LN. The results showed that CS-DEGs were closed tied to icosanoid and prostaglandin biosynthetic process and Inflammatory mediator regulation of TRP channels (Ricciotti and FitzGerald, 2011; Dennis and Norris, 2015). Icosanoid and prostaglandin are both products derived from arachidonic acid. They can mediate bidirectional inflammatory response, which may exert ambiguous influence on the course of LN. TRP channels distributed along the nephron play an important role in renal diseases (Hsu et al., 2007). These findings suggest that CS-DEGs may make a difference in the progression of LN. Then, three hub CS-DEGs genes ALOX5, PTGER2 and PRKCB were identified as biomarkers through machine learning algorithms and they were deemed promising for the new perspective of the diagnosis and treatment for LN. To further explore their effects on LN, GSEA and immune filtration analysis were performed.

ALOX5 (arachidonate 5-lipoxygenase) encodes a pivotal enzyme responsible for the synthesis of proinflammatory leukotrienes from arachidonic acid. ALOX5 participates in cellular senescence by accelerating growth arrest through a p53/p21-dependent pathway independently of telomerase activity (Catalano et al., 2005). A metabolic study containing 20 SLE patients and nine healthy controls found that Leukotriene B4 (LTB4) and 5-Hydroxyeicosatetraenoic acid (5-HETE) were significantly elevated in SLE, which could discriminate SLE patients from normal controls (Wu et al., 2012). Since the last century, a flow of investigation has implied the renal damage of leukotriene, and montelukast, a kind of CysLT1 receptor antagonist, could attenuate oxidative stress, histopathological markers of tissue damage, cytokine release and protect renal function (Rubinstein and Dvash, 2018). Also, LTB4 and its receptor BLT1 were demonstrated to involve in the pathogenesis of glomerulonephritis mediated by immune complex (Shioda et al., 2023). Evidence has shown that interleukin 1β, tumour necrosis factor α, and histamine signaling enhance ALOX5 activity, ultimately triggering reactive oxygen species (ROS)-mediated NF-κB activation, which is consistent with our GSEA result that ALOX5 links to NF-κB signal pathway. These findings potently support the kidney affliction of ALOX5 in the etiology of LN.

PTGER2 (Prostaglandin E Receptor 2) encodes a receptor for prostaglandin E2 (PGE2), namely, EP2, which is another metabolite of arachidonic acid apart from leukotrienes. PGE2 can be generated by almost all types of cells and deeply impact pathophysiological activity by binding to different prostaglandin E receptors EP1, EP2, EP3, and EP4 (Cheng et al., 2021). PTGER2 is an important mediator in cellular senescence. Increasing levels of PGE2 with age impaired glycolysis and restrained the mitochondrial oxygen consumption rate (OCR) in human monocyte-derived macrophages (MDMs), which depended on the EP2 receptor, rather than other receptors. The inhibition of PGE2–EP2 signaling by EP2 agonist leaded to the restoration of energy production in ageing myeloid cells (Minhas et al., 2021). According to this bioinformatic analysis, PTGER2, which encodes EP2, may do harm to the kidney through cellular senescence in the context of LN. But the role of EP2 in renal diseases remains elusive. Some studies demonstrated that stimulation of the EP2 receptor effectively mitigates renal fibrogenesis and relieves chronic kidney failure (Nasrallah et al., 2014; Jensen et al., 2019). While some studies announced that EP2 take part in the podocyte affliction of tensile stress and fluid flow shear stress (FFSS), which is a markable cause for albuminuria in hyperfiltration-mediated kidney damage (Srivastava et al., 2014). The question about whether PTGER2 plays a protective or detrimental role in kidney diseases lacks general consensus and calls for more investigation.

PRKCB (protein kinase C beta) belong to a family of serine- and threonine-specific protein kinases. PRKCB has been reported to connect with various cellular functions, including oxidative stress–induced apoptosis, androgen receptor–dependent transcription regulation, B Cell activation, intestinal sugar absorption, endothelial cell proliferation, energy metabolism and autophagy (Steinberg, 2008; Mehta, 2014). In addition, PRKCB plays a central role in B Cell receptor (BCR)-mediated NF-κB activation and inhibition of PRKCB accelerates cell death in B lymphomas characterized by activated NF-κB (Su et al., 2002). An investigation recruited 60 patients with SLE and 62 healthy controls and examined their expression of PRKCB mRNA in peripheral blood mononuclear cells (Zhu et al., 2018), which found the PRKCB mRNA expression levels of SLE patients were significantly augmented in comparison with those in healthy cases. Notably, the investigation also found the levels of PRKCB mRNA were negatively correlated with SLEDAI and proteinuria. Besides, evidence shows that PRKCB upregulated at the gene expression level in diabetic nephropathy, proven to be a predictor for worsening of kidney disease in subjects with type 2 diabetes (Araki et al., 2006; Langham et al., 2008). Our study confirmed PRKCB could predict the onset of LN with accuracy and was positively correlated with proteinuria in LN patients based on the nephroseq database, which is a bit contradictory with the foreign investigation. In conclusion, it deserves more consideration and investigation focused on the influence of PRKCB on the progression of LN.

To further delve into the roles of ALOX5, PTGER2 and PRKCB in the pathology mechanisms of LN, the single-gene GSEA analysis was performed. The results indicated the functions of three genes were centered on “B Cell receptor signaling pathway” and “NF−kappa B signaling pathway”. Aging extensively reshapes the generation and function of B Cells, which contributes to the pathogenesis of several autoimmune diseases such as SLE, rheumatoid arthritis, scleroderma. Crohn disease and Sjögren syndrome (Cancro, 2020). A clinical trial showed that in SLE patients treated with belimumab, an inhibitor of B-lymphocyte stimulator (BLyS) which exceptionally attenuate the risk of lupus nephritis flare and eGFR decline, the loss of cells consistent with the age-associated B Cell (ABC) phenotype was correlated with therapeutic response. Conclusive evidence illustrated that senescent cells drive production of reactive oxygen species (ROS) so as to activate NF-κB signaling pathway (Nelson et al., 2018). NF-kappa B signaling pathway participates in the initiation of inflammatory response and promotes the onset of diverse autoimmune diseases including LN (Yu et al., 2020). The upregulated expression of molecules related to the NF-κB signaling pathway in the kidneys of MRL/lpr mice indicated that activation of the NF-κB pathway is an important factor of kidney damage in SLE and may be responsible for lupus activity (Li X. et al., 2022).

LN is a multi-organ autoimmune disease with complex microimmune environment. The immune filtration analysis of immune cell percentage between LN groups and control groups showed that LN patients were characterized by increased activated NK cells, monocytes, and decreased resting NK cells, Tregs, significantly associated with three biomarkers. Along with the decline in NK cell cytotoxicity with age declared by some reports (Kucuksezer et al., 2021; Brauning et al., 2022), in SLE patients, the cytotoxic activities of NK cells were also demonstrated to be suppressed (Erkeller-Yuksel et al., 1997; Riccieri et al., 2000; Park et al., 2009). Compared with healthy controls, the CD56dimNK cells in SLE patients produced more IFN-γ and displayed relatively activated phenotypic characteristics, including significant increases in NKp44, NKp46, and CD69 and decreased expression of CD16 and CD158a/h/g (Liu et al., 2021). Though several studies reported that activated NK cells in the kidney may lead to inducing and maintaining inflammation in LN (Spada et al., 2015a; Spada et al., 2015b), data in the literature with respect to the role of NK cells in LN is scarce. The amount of monocyte is elevated in SLE patients, and in particular, CD16^+^ monocytes are infiltrated in the kidney of LN patients, proven to involve in tissue damage and disease activity (Yoshimoto et al., 2007; Barrera García et al., 2016; Kuriakose et al., 2019). The pathological relevance is partly owing to the reason that CD16^+^ monocytes can bind immobilized immune complexes (ICs) in renal blood vessels and glomeruli, promoting their extravasation into the inflamed kidney tissue (Olaru et al., 2018). Tregs are essential for the homeostasis of the immune system by preventing abnormal activation of the immune system and maintaining autoimmune tolerance (Li Y. et al., 2022; Li et al., 2023). Numerous researches highlighted the vital contribution of suppressed regulatory T Cells to the progress of LN, which provide a novel therapeutic approach targeting Treg cells for the control of LN (Venkatadri et al., 2021; Tsai et al., 2023).

Our DGIdb analysis identified ALOX5 as the most frequently targeted gene among the three hub CS-DEGs, followed by PTGER2. Notably, nine compounds demonstrated high interaction scores (>3), including EVATANEPAG, OMIDENEPAG, OMIDENEPAG ISOPROPYL, TAPRENEPAG ISOPROPYL, SIMENEPAG ISOPROPYL, PF-4191834, ATRELEUTON, CHEMBL502496, and ZILEUTON. Of these, five targeted PTGER2 while three acted on ALOX5. This pharmacological profiling suggests both ALOX5 and PTGER2 represent promising therapeutic targets for further investigation in lupus nephritis. The predominance of high-scoring compounds targeting these two genes underscores their potential clinical relevance and warrants future experimental validation.

There remain several limitations of our study. First, the research data from public database only contains expression levels of genes and disease state of each sample. We cannot assess the connection between gene expression levels and clinical features such as the activity of LN, serum creatine levels and proteinura. The lack of large scale real-world clinical information limits the extensive validation of the predictive instrument. Second, additional molecular biology experiments or animal experiments are required to further validate the importance of cellular senescence-related genes in LN and explore their potential pathogenic mechanisms. Third, different pathological types of LN present different prognosis, which lead to different treatment strategies, so investigation concentrated on cellular senescence among various pathological types of LN are needed.

This study explored the potential relationship between cellular senescence and LN, and identified three hub CS-DEGs as biomarkers playing key roles in LN. Genes including ALOX5, PTGER2, and PRKCB were deemed promising for the new perspective of the diagnosis and treatment for LN.

## Data Availability

The datasets presented in this study can be found in online repositories. The names of the repository/repositories and accession number(s) can be found in the article/Supplementary Material.
