# Supplementary material for: Identification of cellular senescence-related genes as biomarkers for lupus nephritis based on bioinformatics
Source: Front Genet. 2025 Apr 11;16:1551450. doi: 10.3389/fgene.2025.1551450 (PMC12021929; doi:10.3389/fgene.2025.1551450)
Supplement: Supplementary file 1 [file Table1.docx]

Supplementary appendix to the manuscript

Table S1. Cellular senescence - related genes

| E2F1 | E2F3 | NF1 | PTEN | ABI3 | ABL1 |
| --- | --- | --- | --- | --- | --- |
| AKT3 | ARG2 | ARNTL | ATM | ATR | B2M |
| BCL2 | BCL6 | BGLAP | BMPR1A | BRCA2 | CALR |
| CDK1 | CDK2 | CDK6 | CDKN1A | CDKN1B | CDKN2A |
| CDKN2B | CGAS | CHEK1 | CHEK2 | CITED2 | CTC1 |
| DNAJA3 | ECRG4 | EEF1E1 | ERCC1 | FBXO5 | FOXM1 |
| FZR1 | H2AX | HLA-G | HMGA1 | HMGA2 | HRAS |
| ID2 | IGF1R | ING2 | KAT5 | KAT6A | KIR2DL4 |
| LIMS1 | LMNA | MAGEA2 | MAGEA2B | MAP2K1 | MAP2K3 |
| MAP2K4 | MAP2K6 | MAP2K7 | MAP3K3 | MAP3K5 | MAPK10 |
| MAPK11 | MAPK14 | MAPK8 | MAPK9 | MAPKAPK5 | MARCHF5 |
| MIF | MIR10A | MIR146A | MIR17 | MIR188 | MIR20B |
| MIR21 | MIR217 | MIR22 | MIR342 | MIR34A | MIR370 |
| MIR543 | MIR590 | MME | MNT | MORC3 | NEK4 |
| NEK6 | NOX4 | NPM1 | NSMCE2 | NUAK1 | NUP62 |
| OPA1 | PAWR | PDCD4 | PLA2R1 | PLK2 | PML |
| PNPT1 | PRELP | PRKCD | PRMT6 | RBL1 | ROMO1 |
| RSL1D1 | RWDD1 | SERPINE1 | SIRT1 | SLC30A10 | SMC5 |
| SMC6 | SOD1 | SPI1 | SRF | TBX2 | TBX3 |
| TERC | TERF2 | TERT | TP53 | TP63 | TWIST1 |
| ULK3 | VASH1 | WNT1 | WNT16 | WRN | YBX1 |
| YPEL3 | ZKSCAN3 | ZMIZ1 | ZMPSTE24 | ZNF277 | ADRA1A |
| AVPR1A | AVPR1B | CISD2 | COMP | COQ7 | CRYAB |
| DDC | EDN1 | EDNRA | GBA | GHRHR | GNA11 |
| GNA12 | GNA13 | HELT | HYAL2 | IDE | INHBB |
| INPP5D | LEP | LRRK2 | MSH2 | MSH6 | NR5A1 |
| PRDM2 | RAD54B | RAD54L | RNF165 | SEC63 | SERP1 |
| SLC1A2 | TFCP2L1 | TH | TREX1 | ACD | AGO1 |
| AGO3 | AGO4 | ANAPC1 | ANAPC10 | ANAPC11 | ANAPC15 |
| ANAPC16 | ANAPC2 | ANAPC4 | ANAPC5 | ANAPC7 | ASF1A |
| BMI1 | CABIN1 | CBX2 | CBX4 | CBX6 | CBX8 |
| CCNA1 | CCNA2 | CCNE1 | CCNE2 | CDC16 | CDC23 |
| CDC26 | CDC27 | CDK4 | CDKN2C | CDKN2D | CEBPB |
| CXCL8 | E2F2 | EED | EHMT1 | EHMT2 | EP400 |
| ERF | ETS1 | ETS2 | EZH2 | FOS | H1-0 |
| H1-1 | H1-2 | H1-3 | H1-4 | H1-5 | H2AB1 |
| H2AC14 | H2AC20 | H2AC4 | H2AC6 | H2AC7 | H2AC8 |
| H2AJ | H2AZ1 | H2AZ2 | H2BC1 | H2BC10 | H2BC11 |
| H2BC12 | H2BC13 | H2BC14 | H2BC15 | H2BC17 | H2BC21 |
| H2BC3 | H2BC4 | H2BC5 | H2BC6 | H2BC7 | H2BC8 |
| H2BC9 | H2BS1 | H2BU1 | H3-3A | H3-3B | H3-4 |
| H3C1 | H3C10 | H3C11 | H3C12 | H3C13 | H3C14 |
| H3C15 | H3C2 | H3C3 | H3C4 | H3C6 | H3C7 |
| H3C8 | H4-16 | H4C1 | H4C11 | H4C12 | H4C13 |
| H4C14 | H4C15 | H4C2 | H4C3 | H4C4 | H4C5 |
| H4C6 | H4C8 | H4C9 | HIRA | ID1 | IFNB1 |
| IGFBP7 | IL1A | IL6 | JUN | KDM6B | LMNB1 |
| MAP4K4 | MAPK1 | MAPK3 | MAPK7 | MAPKAPK2 | MAPKAPK3 |
| MDM2 | MDM4 | MINK1 | MIR24-1 | MIR24-2 | MOV10 |
| MRE11 | NBN | NFKB1 | PHC1 | PHC2 | PHC3 |
| POT1 | RAD50 | RB1 | RBBP4 | RBBP7 | RELA |
| RING1 | RNF2 | RPS27A | RPS6KA1 | RPS6KA2 | RPS6KA3 |
| SCMH1 | SP1 | STAT3 | SUZ12 | TERF1 | TERF2IP |
| TFDP1 | TFDP2 | TINF2 | TNIK | TNRC6A | TNRC6B |
| TNRC6C | TXN | UBA52 | UBB | UBC | UBE2C |
| UBE2D1 | UBE2E1 | UBE2S | UBN1 | VENTX | ALDOC |
| ENO1 | G6PD | GAPDH | HK1 | LDHA | PGK1 |
| PKM | PRKAA1 | AADAT | ACMSD | AFMID | AHR |
| EIF2AK1 | EIF2AK4 | FOXO1 | HAAO | IDO1 | IDO2 |
| IFNG | IL1R2 | KLF5 | KMO | KYNU | NOS1 |
| QPRT | TDO2 | TLR4 | TNF | CCL27 | ELAVL1 |
| GOT1 | GOT2 | IL1B | MDH1 | MDH2 | NAMPT |
| NMNAT2 | PARP1 | SCO2 | SIRT2 | SIRT3 | SIRT5 |
| SLC2A1 | SLC2A4 | ADCY3 | ALOX12 | ALOX15 | ALOX15B |
| ALOX5 | ALOX5AP | CYSLTR1 | GNAI1 | GNAQ | GNAS |
| IGFBP5 | LTA4H | LTC4S | PLA2G4A | PLCB1 | PRXL2B |
| PTGDS | PTGER1 | PTGER2 | PTGER3 | PTGER4 | PTGES |
| PTGIS | PTGS1 | PTGS2 | TBXAS1 | ASAH1 | CERS2 |
| CERS4 | DEGS1 | GLB1 | KDSR | MTOR | PRKCA |
| PRKCB | RPP38 | S1PR1 | S1PR2 | S1PR5 | SGMS2 |
| SGPP1 | SMPD3 | SPHK1 | SPTLC1 | UGCG | BRAF |
| FH | ME1 | ME2 | PDHA1 | PDK1 | PDP2 |

Table S2. Summary of the datasets on lupus nephritis utilized in this study

| **Dataset** | **platform** | **Number of LN samples** | **Number of Controls** | **Group** |
| --- | --- | --- | --- | --- |
| GSE32591 | GPL14663 | 32 | 14 | Training set |
| GSE127797 | GPL24299 | 41 | 0 | Training set |
| GSE104948 | GPL24120, GPL22945 | 32 | 21 | Training set |
| GSE180393 | GPL19983 | 15 | 9 | Validation set |

Table S3. 20 CS-DEGs

| **Gene** | **logFC** | **AveExpr** | **t** | **P.Value** | **adj.P.Val** | **B** |
| --- | --- | --- | --- | --- | --- | --- |
| PTGER2 | 1.432428 | 6.269375 | 16.25119 | 3.68E-34 | 6.13E-32 | 67.12882 |
| HYAL2 | 0.94658 | 8.89553 | 11.31991 | 1.49E-21 | 5.23E-20 | 38.27118 |
| ALOX5 | 0.887873 | 5.862267 | 10.79284 | 3.49E-20 | 9.73E-19 | 35.14287 |
| PTGS1 | 0.864959 | 6.853746 | 10.23872 | 9.44E-19 | 2.20E-17 | 31.8693 |
| PLA2G4A | 0.917715 | 4.515078 | 10.15696 | 1.53E-18 | 3.48E-17 | 31.38817 |
| MAP4K4 | 0.730485 | 7.314815 | 9.553933 | 5.38E-17 | 1.01E-15 | 27.86019 |
| PRKCB | 0.750491 | 5.666431 | 9.539322 | 5.86E-17 | 1.09E-15 | 27.77523 |
| TWIST1 | 0.631678 | 5.317026 | 9.318929 | 2.13E-16 | 3.70E-15 | 26.49713 |
| IDO1 | 1.5073 | 6.689963 | 9.06972 | 9.08E-16 | 1.45E-14 | 25.06051 |
| RPS6KA3 | 0.687536 | 6.370167 | 8.949051 | 1.83E-15 | 2.81E-14 | 24.3685 |
| S1PR1 | 0.686964 | 8.077974 | 8.751368 | 5.71E-15 | 8.20E-14 | 23.24039 |
| EZH2 | 0.646728 | 4.729593 | 8.307488 | 7.19E-14 | 8.98E-13 | 20.73553 |
| TBX3 | 0.799929 | 7.39433 | 8.09965 | 2.32E-13 | 2.73E-12 | 19.57776 |
| PTGER4 | -0.51341 | 8.141051 | -7.89566 | 7.26E-13 | 7.90E-12 | 18.45189 |
| LMNB1 | 0.523951 | 4.839025 | 7.580956 | 4.13E-12 | 4.02E-11 | 16.73707 |
| SGPP1 | -0.55433 | 5.54174 | -7.39712 | 1.12E-11 | 1.03E-10 | 15.74888 |
| VASH1 | 0.627219 | 7.362483 | 7.169535 | 3.84E-11 | 3.27E-10 | 14.54044 |
| PLK2 | 0.841521 | 6.883892 | 6.432833 | 1.81E-09 | 1.19E-08 | 10.7566 |
| PTGS2 | 0.64909 | 4.617765 | 6.111461 | 9.08E-09 | 5.43E-08 | 9.175255 |
| IL1B | 0.57807 | 6.407711 | 5.562157 | 1.29E-07 | 6.40E-07 | 6.584656 |

Table S4. hub CS-DEGs identified by three machine learning methods

| SVM-REF | LASSO | RF |
| --- | --- | --- |
| PTGER4  HYAL2  PTGER2  ALOX5  PLA2G4A  PTGS1  IDO1  LMNB1  IL1B  SGPP1  PLK2  PRKCB | PLK2  TBX3  VASH1  HYAL2  EZH2  LMNB1  MAP4K4  RPS6KA3  IDO1  ALOX5  PLA2G4A  PTGER2  PTGER4  PTGS1  PTGS2  PRKCB  S1PR1  SGPP1 | PLA2G4A  PTGER2  HYAL2  PTGS1  ALOX5  RPS6KA3  PRKCB  EZH2  MAP4K4 |
